# Blood pressure variability and cognitive dysfunction: A systematic review and meta‐analysis of longitudinal cohort studies

**DOI:** 10.1111/jch.14310

**Published:** 2021-06-21

**Authors:** Tzu‐Jung Chiu, Jiunn‐Tyng Yeh, Chi‐Jung Huang, Chern‐En Chiang, Shih‐Hsien Sung, Chen‐Huan Chen, Hao‐Min Cheng

**Affiliations:** ^1^ Department of Medicine National Yang Ming Chiao Tung University College of Medicine Taipei Taiwan; ^2^ Center for Evidence‐Based Medicine Taipei Veterans General Hospital Taipei Taiwan; ^3^ General Clinical Research Center Taipei Veterans General Hospital Taipei Taiwan; ^4^ Division of Cardiology, Department of Internal Medicine Taipei Veterans General Hospital Taipei Taiwan; ^5^ Institute of Public Health and Community Medicine Research Center National Yang Ming Chiao Tung University College of Medicine Taipei Taiwan; ^6^ Department of Medical Education Taipei Veterans General Hospital Taipei Taiwan

**Keywords:** blood pressure variability, cognitive dysfunction, cohort studies, dementia, meta‐analysis

## Abstract

The variability of blood pressure (BPV) has been suggested as a clinical indicator for cognitive dysfunction, yet the results from clinical studies are variable. This study investigated the relationship between BPV and the risk of cognitive decline or dementia. Bibliographic databases, including PubMed, Scopus, and Embase, were searched systematically for longitudinal cohort studies with BPV measurements and neuropsychological examinations or dementia diagnosis. A traditional meta‐analysis with subgroup analysis, and a further dose‐response meta‐analysis were conducted. Twenty cohort studies with 7 924 168 persons were included in this review. The results showed that a higher systolic BPV (SBPV), when measured with the coefficient of variation (SBP‐CV) or standard deviation (SBP‐SD), was associated with a higher risk of all‐cause dementia diagnosis but not incidence of cognitive decline on neuropsychological examinations. In subgroup analysis, the effect was more prominent when using BPV of shorter timeframes, during shorter follow‐ups, or among the elderly aged more than 65 years. No dose‐response relationship could be found. Our study suggested possible positive associations between SBPV and the risk of dementia. Further studies are required to validate these findings.

## 1 INTRODUCTION

Dementia is a common neurologic syndrome manifested by an abnormal decline in cognitive function affecting over 47 million people worldwide.^1^ Over the past decade, the importance of vascular risk factors, especially hypertension, in dementia has emerged from epidemiological and biomedical studies.^2^, ^3^ Elevated blood pressure (BP) damages the endothelia and increases the risk of stroke; this is proposed to contribute to the multifaceted pathogenesis of dementia.^4^, ^5^

Apart from elevated BP, BP variability (BPV) has been proposed as another important vascular risk factor.^6^, ^7^ BPV is a collective term depicting the homeostasis of BP in response to internal and external stimulations. It encompasses a range of estimation of the variation of systolic BP (SBP), diastolic BP (DBP), or pulse pressure measured within different timeframes (eg, very short‐term, short‐term, mid‐term, and long‐term) using different methods for measurement (eg, beat‐to‐beat, ambulatory, day‐to‐day, and visit‐to‐visit BP measurements) and characterized by different patterns (eg, nocturnal, postural, and postprandial).^6^ Different statistical indices (eg, standard deviation, coefficient of variation, and variation independent of the mean) were calculated to estimate the fluctuation of the BP. BPV is associated with organ damage, stroke, cardiovascular events, and mortality even after adjusting for average BP, indicating its independent role as a vascular risk factor.^8^, ^9^

Several population‐based studies have investigated the relationship between BPV and cognitive decline or dementia; however, the results were inconsistent.^10^, ^11^ In this systematic review and meta‐analysis, we aimed to summarize the current evidence on associations between BPV and the incidence of cognitive decline or dementia.

## 2 METHODS

### 2.1 Protocol and registration

This study was conducted and reported according to the Preferred Reporting Items for Systemic Review and Meta‐Analysis Statement (PRISMA) 2020 guideline.^12^ The research protocol was published in the International Prospective Register of Systematic Reviews (PROSPERO) with the registration number CRD42020190429.

### 2.2 Eligibility criteria

#### 2.2.1 Types of studies

Cohort studies with longitudinal follow‐up for the incidence of cognitive decline or dementia risk were used including prospective, retrospective, and post‐hoc or subgroup analyses from a larger cohort or randomized controlled trial, with accessible full‐text content in English.

#### 2.2.2 Types of participants

No limitations on age or baseline health status were applied, except for the diagnosis of dementia at baseline. The participants had both BPV measurements and global cognitive decline or dementia monitoring in a longitudinal manner.

#### 2.2.3 Types of exposures

Any measure of SBPV or DBPV obtained from ambulatory, home, or visit‐to‐visit BP monitoring was eligible. The BPV indices included the following three categories: overall variability, variability between consecutive visits, and the extremes in values on a single visit. Overall variability was assessed using standard deviation (SD), coefficient of variation (CV), and variance independent of the mean (VIM). Variability between consecutive visits was assessed with average real variability (ARV), and the extremes in values on a single visit was assessed with full range (difference between the maximum and the minimum).^13^ Timeframes of BPV were classified into short‐term (as measured with ambulatory BP), mid‐term (with day‐to‐day BP), and long‐term (with visit‐to‐visit BP) . Nocturnal and orthostatic BP variability were not included in the present systematic review.^14^

#### 2.2.4 Types of outcomes

The primary outcome was the incidence of all common types of dementia, including Alzheimer's disease and vascular dementia, verified by licensed physicians or related professionals or medical records. The secondary outcome was the incidence of cognitive decline across any period, obtained by standardized neuropsychological tests, including Mini‐Mental State Examination (MMSE), Montreal Cognitive Assessment (MoCA), Cambridge Cognition Examination (CAMCOG), etc., at least twice.

### 2.3 Data sources and search strategy

Three bibliographic databases, PubMed, Scopus, and Embase, were searched on May 11, 2021, without limitations on the publication date. Bibliographies of the included studies and relevant publications were also manually searched for eligible studies. The search string used was as followed, (“blood pressure variability” OR “beat‐to‐beat” OR “24‐hour blood pressure monitoring” OR “visit‐to‐visit” OR “ambulatory blood pressure monitoring” OR “home blood pressure monitoring”) AND (“dementia” OR “Alzheimer's disease” OR “vascular dementia” OR “Frontotemporal Dementia” OR “cognitive impairment” OR “Mini‐Mental State Examination” OR “cognitive function” OR “cognitive testing” OR “neuropsychological testing” OR “memory”).

### 2.4 Study selection

After removing duplicate studies, two reviewers (T.J.C. and J.T.Y.) independently screened the titles and abstracts. The full text was retrieved for further assessment. The reviewers then independently assessed the full articles according to the inclusion/exclusion criteria. Disagreements were resolved by consensus and by consulting with a third reviewer (HMC).

### 2.5 Data extraction

The two reviewers independently extracted study characteristics and outcome data from the included studies. We only extracted the numbers derived from the fully‐adjusted model in each study. The variables adjusted were listed in Table 1. Discrepancies were resolved by discussion with a third reviewer (HMC).

**TABLE 1:** Study characteristics

Reference	Database	Population description	No. of persons	Follow up duration for cognitive performance	BPV measurement modality^a^ & time point	Age(yr,)^b^	Male(%)	Comorbidity^c^	BPV metrics	Outcome	Definition of outcome^d^	Adjustment factors	Main finding^e^
Alperovitch 2014^27^	Three‐City (3C) Study	non‐institutionalized persons aged 65 years and older	6506	8 yr	office BP or home BP; baseline, 2 yr, 4 yr	73.7(5.2)	38	HTN (%) 76.50 BMI (kg/m2) 25.61 Current or past smoker (%) 38.07 Current or past drinker (%) 83.52 Mean total chol. (mmol/l) 5.81 DM (%) 9.28 History of vascular event (%)^g^ 8.48 Depression (%) 12.40	SBP‐CV, DBP‐CV	dementia risk	by DSM‐IV; all‐cause dementia: diagnosed by DSM‐IV (+)	sex, study center, education, DM^f^, history of vascular diseases^f^, antihypertensive drug at baseline^f^, and mean BP	SBP‐CV & DBP‐CV positively associated with dementia risk
Bohm 2015^20^	ONTARGET, TRANSCEND	patients aged > = 55 years with certain comorbidities (without symptomatic heart failure at entry and with a history of CAD, PAD, prior TIA or stroke or DM complicated by organ damage)	24593	5 yr	office BP; baseline, 6wk, 6mon, every 6mon till last MMSE (2 or 3–5 yr)	66.0 (7.0)	72.5	HTN (%) 69 BMI (kg/m2) 28.2 Current or past smoker (%) 63.8 Current or past drinker (%) 41.4 DM (%) 46.4 History of MI (%) 49.3 History of stroke/TIA (%) 22.6 depression (%) 21	SBP‐CV	Incidence of cognitive decline	by MMSE; 1. cognitive dysfunction: < = 24pts at 2 yr or 3–5 yr; 2. cognitive decline: > = 5pts decrease; 3. cognitive deterioration: > = 1pt decrease/yr or < = 24pts	MMSE value at baseline, DBPV^f^, age^f^, BMI^f^, eGFR (MDRD)^f^, sex^f^, ethnicity^f^, physical activity^f^, formal education^f^, alcohol consumption^f^, history of stroke and stroke during study conduct^f^, history of DM and new DM during study conduct^f^, concomitant medications with aspirin^f^, beta blockers, diuretics, nitrates, statins, and hypoglycemics	SBP‐CV positively associated with incidence of cognitive decline (no assessment on DBPV)
Epstein 2013^3^	ADNI	volunteers aged 55∼90 years (including Petersen criteria MCI, AD, and healthy control)	428	3 yr	office BP; baseline, 6mon, 12mon, 18mon, 24mon, 36mon	75.2 (6.4)	59.8	BMI at 3 yr (kg/m2) 26 Current or past smoker (%) 38.8 DM at 3 yr (%) 8.6 High chol. (%) 72.2 History of vascular disease (%) 14 Depression (%) 21.7	SBP‐CV, SBP‐SD, DBP‐CV, DBP‐SD	Incidence of cognitive decline	by ADAS‐COG, MMSE, CDR, etc.; cognitive score, at 36mon: pts at the 36mon	baseline cognitive scores, age^f^, years of education^f^, sex^f^, presence of apolipoprotein E ε4 allele^f^, and vascular disease^f^, BMI, and depression at 36 months^f^	SBP‐SD & SBP‐CV positively associated with incidence of cognitive decline (no association regarding DBP‐SD & DBP‐CV with incidence of cognitive decline)
Geng 2017^19^	N/A (original prospective cohort)	patients with acute ischemic stroke	708	1 yr (3mon as outcome)	office BP; baseline∼7day, per 4hr	63.1 (10.0)	54.1	HTN (%) 88.1 BMI (kg/m2) 25.7 Current smoker (%) 29.8 Current drinker (%) 21.5 Hyperlipidemia (%) 56.1 DM (%) 22.7 CAD (%) 13.6 Stroke (%) 100 History of TIA (%) 16.1	SBP‐CV, SBP‐SD, DBP‐CV, DBP‐SD	Incidence of cognitive decline	by MoCA; “PSCI”: education < 12 yr + MoCA < = 25pts, or education > 12 yr + MoCA < = 26pts	age^f^, sex, education degree (less than 12 years)^f^, HTN^f^, SBP and DBP on admission^f^, CIV and location of infarction (classified as cortex, cortex‐subcortical, brain stem, and cerebellum), NIHSS^f,^ and thrombolytic therapy^f^	SBP‐CV positively associated with incidence of cognitive decline (no association regarding DBP‐CV & DBP‐SD with incidence of cognitive decline)
													
Haring 2019^25^	WHIMS‐MRI	Postmenopausal healthy women (without CVD, DM, HTN, or current smoking at baseline)	558	9‐11 yr	office BP; baseline, per 1 yr f/u till last 3MSE	78.2 (3.6)	0	HTN (%) 0 Current smoker (%) 0 DM (%) 0 CAD (%) 0 Stroke (%) 0	SBP‐SD, SBP‐SDreg^h^, DBP‐SD DBP‐SDreg^h^	Incidence of cognitive decline	by 3MSE; Mean 3MSE annual change: pts change per 1 yr	age, education, presence of APOE ε4 allele, hormone therapy randomization arm, and mean BP	No association between either SBP‐SD or DBP‐SD and incidence of cognitive decline
Kim 2021^24^	PICASSO‐COG Sub‐study	patients with non‐cardioembolic ischemic stroke or TIA within 180 days who had prior ICH or multiple cerebral microbleeds	1240	4 yr	office BP; 1mon∼4 yr, per 3 mon	64.6 (10.8)	64.1	HTN (%) 89.5 Smoking (%) 47.6 DM (%) 30.9 Hyperlipidemia 43.2 CAD (%) 4.1 Stroke (%) 94.8 TIA (%) 5.2	SBP‐CV, SBP‐SD, SBP‐VIM, SBP‐SDreg^h^	Incidence of cognitive decline	by MMSE and MoCA; cognitive performance: total pts in MMSE and MoCA	age, sex, educational years, probucol treatment, baseline NIHSS score, baseline cognition test scores, DM, index of high risk of ICH, and mean SBP	SBP‐SD & SBP‐VIM & SBP‐SDreg possitively associated with cognitive decline.
Liu 2015^26^	N/A (original prospective cohort)	oldest old from geriatric practices and community‐dwelling	232	baseline, f/u per 3mon final visit (ave. 2.3 yr)	home BP; baseline, 1day, 2day, 3day, 4day, 5day, 6day, 1wk; day & night	84.35 (2.52)	25.4	BMI (kg/m2) 23.49 Current or past smoker (%) 9.05 Alcohol (units per week) 0.41 Total chol. (mmol/l) 4.47 DM (%) 0 History of stroke (%) 0	SBP‐CV	Incidence of cognitive decline	by MMSE; Pt change percentage, baseline & final visit: (final‐baseline)/baseline	baseline MMSE score, baseline WMH fraction, age, sex, baseline BMI, baseline office BP, baseline blood lipid and glucose, education, smoking and alcohol consumption	SBP‐CV positively associated with incidence of cognitive decline (no assessment on DBPV)
Ma 2019^29^	Rotterdam study	dementia free participants	5273	26 yr (20‐22 yr as outcome)	office BP; baseline‐5 yr, 5–7 yr, 9–11 yr, 13–15 yr, 20–22 yr (total 0∼26 yr)	67.6 (8.0)	41.9	HTN (%) 58.8 Overweight/obese (%) 61.8 BMI (kg/m2) 26.3 Current or past smoker (%) 63.7 Current drinker (%) 80.8 Total chol. (mmol/l) 6.7 DM (%) 6.7 CHD (%) 7.1 Stroke (%) 1.9	SBP‐CV, SBP‐SD, SBP‐ARV, DBP‐CV, DBP‐SD, DBP‐ARV	dementia risk	by DSM‐III‐R, NINCDS‐ADRDA, NINDS‐AIREN; 1. all‐cause dementia, lag0/5/10/15 :min 0/5/10/15 yr interval between SBPV & DSM‐III‐R(+) 2. AD, lag0/5/10/15: min 0/5/10/15 yr interval between SBPV & DSM‐III‐R(+) 3. VaD, lag0/5/10/15: min 0/5/10/15 yr interval between SBPV & DSM‐III‐R(+)	age, sex, education level, APOE genotype, smoking habits, alcohol consumption, BMI, lipid levels, history of DM and CVD, and antihypertensive medication	SBP‐ARV & DBP‐ARV positively associated with dementia risk
Ma 2021^36^	ADRCs Program of the National Institute on Aging through NACC	dementia free adults, aged > = 50 y/o	13284	14 yr	Office BP; baseline∼14 yr, per 1 yr	72 (9)	41.2	BMI (kg/m2) 27.3 Current or past smoker (%) 43.0 HTN (%) 69.0 DM (%) 12.1 History of CVD (%) 11.2 TIA/stroke (%) 5.2	SBP‐CV, SBP‐RMSE^i^, DBP‐CV, DBP‐RMSE^i^	Incidence of cognitive decline	by CDR; cognitive deterioration: progression of cognitive status, specifically with CDR‐SOB.	age, sex, mean BP, rate of change in BP, antihypertensive medication use, education level, APOE genotype, smoking habits, weight status, history of DM, baseline CDR score, and years of follow‐up	SBP‐CV & SBP‐RMSE & DBP‐CV & DBP‐RMSE possitively associated with cognitive decline.
Matsumoto 2014^15^	The Ohasama Study	community‐based population	485	f/u visit per 4 yr from baseline (median 7.8 yr)	home BP; baseline∼4wk, daily	63.3 (4.7)	28	BMI (kg/m2) 23.8 Current or past smoker (%) 11 Current or past drinker (%) 30 Hypercholesterolemia (%) 41 DM (%) 12 History of CVD (%) 8	SBP‐SD, DBP‐SD	Incidence of cognitive decline	by MMSE; cognitive decline: MMSE < 24pts	sex, age, history of CVD, low level of education, baseline MMSE score < 27, follow‐up duration, and home SBP	SBP‐SD positively associated with incidence of cognitive decline (no association regarding DBP‐SD with incidence of cognitive decline)
McDonald 2017^16^	N/A (original prospective cohort)	community‐dwelling older persons	205	5 yr	ambulatory BP; 0∼24hr, daytime per 30 min, nighttime per 60 min	72 (68‐77) (median, IQR)	58	HTN (%) 42 Stroke (%) 11	SBP‐CV, DBP‐CV	Incidence of cognitive decline	by MMSE, CAMCOG; cognitive change: pts difference between baseline & 5 yr	age, sex, years in full‐time education, mean BP, use of cardioactive medication, and history of stoke	SBP‐CV positively associated with incidence of cognitive decline (no association regarding DBP‐CV with incidence of cognitive decline)
Middelaar 2017^39^	preDIVA trial	community‐dwelling older people (aged 70 to 78 years)	dementia: 2275 cognitive decline: 2305	8 yr	office BP; baseline, 2 yr, 4 yr, 6 yr, 8 yr	74.2 (2.5)	44.8	BMI (kg/m2) 27.5 Current smoker (%) 12.1 DM (%) 19 LDL chol. (mmol/l) 3.1 History of CVD (%) 33	SBP‐CV, SBP‐SD, SBP‐VIM, SBP‐ARV, SBP‐full range, DBP‐CV, DBP‐SD, DBP‐VIM, DBP‐ARV, DBP‐full range	dementiarisk, incidence of cognitive decline	by DSM‐IV, MMSE; 1. all‐cause dementia: DSM‐IV(+), 2. cognitive decline: average MMSE pts declined per visit	sex, age, low educational level (no education or primary education only), obesity (BMI ≥30 kg/m^2^), LDL chol., smoking and DM	SBP‐CV positively associated with incidence of cognitive decline (no association between BPV and dementia risk)
Oishi 2017^28^	The Hisayama study (original prospective cohort)	Japanese elderly without dementia, > = 60 y/o	1674	5 yr	home BP; baseline∼28day, daily in daytime	71 (7)	44.1	BMI (kg/m2) 23.1 Current or past smoker (%) 13 Current or past drinker (%) 42.4 Total chol. (mmol/l) 5.37 DM (%) 21.4 History of CVD (%) 8	SBP‐CV, SBP‐SD, SBP‐VIM, SBP‐ARV, SBP‐full range, DBP‐CV, DBP‐SD, DBP‐VIM, DBP‐ARV, DBP‐full range	dementia risk	by DSM‐III‐R, NINCDS‐ADRDA, NINDS‐AIREN; 1. all‐cause dementia: DSM‐III‐R(+), 2. AD: NINCDS‐ADRDA(+), 3.VaD: NINDS‐AIREN(+)	age, sex, education level, use of antihypertensive agents, ECG abnormalities, DM, serum total chol., BMI, history of CVD, smoking habit, alcohol intake, regular exercise, and mean home SBP for 4 weeks	SBP‐CV & DBP‐CV positively associated with dementia risk, including all‐cause dementia, AD, and VaD
Okonkwo 2011^21^	N/A (original prospective cohort)	community‐dwelling individual with established history of CVD	172	3 yr	office BP; baseline∼2hr, per 10 min	69.18 (7.61)	62	HTN (%) 72.9 Hypercholesterolemia (%) 41.4 DM (%) 21.5 History of CAD/MI (%) 77.5	SBPV, DBPV (statistic indices not mentioned)	Incidence of cognitive decline	by non‐global cognitive test (eg, Trail Making Test (parts A and B, TMT‐ A&B), Letter Cancellation test, Stroop test, Controlled Oral Word Association (COWA) test, etc.); decline in Attention‐Executive‐Psychomotor function, baseline & 3 yr: decrease in z‐transformed pts of baseline & 3 yr"	age, baseline score on the Attention‐Executive‐Psychomotor composite	SBPV & DBPV positively associated with dementia risk
Qin 2016^17^	China Health and Nutrition Survey (CHNS)	community‐dwelling Chinese individuals	976	13 yr	office BP; baseline, 2 yr, 6 yr, 9 yr	63.4 (6.7)	48.2	BMI (kg/m2) 22.2 Current or past smoker (%) 47.0 DM (%) 2.3 History of MI (%) 1.3 History of stroke (%) 1.9	SBP‐CV, SBP‐SD, SBP‐VIM, DBP‐CV, DBP‐SD, DBP‐VIM	Incidence of cognitive decline	by Telephone Interview for Cognitive Status‐modified; global composite cognitive score: sum of all, 0∼31pts	age, sex, education (highest level of education attained primary versus less), time (years since baseline), urbanization index^f^, ever smoking^f^, physical activity (categorical variables in tertiles)^f^, antihypertensive treatment^f^, mean SBP^f^, and their time interactions	SBP‐SD positively associated with incidence of cognitive decline; DBP‐SD only positively associated with incidence of cognitive decline in certain subgroup
Rouch 2020^22^	S.AGES Cohort	noninstitutionalized patients aged 65 years and older, with specific comorbidities (one of the following conditions: chronic pain, type 2 DM, or atrial fibrillation)	3491	3 yr	office BP; baseline∼3 yr, per 6 mon	77.7 (6.2)	43.5	BMI (kg/m2) 27.9 Current or past smoker (%) 25.7 DM (%) 40.9 Dyslipidemia (%) 45 CAD (%) 11 TIA/stroke (%) 6.8″	SBP‐CV, SBP‐SD, SBP‐ARV, SBP‐VIM, DBP‐CV, DBP‐SD, DBP‐ARV, DBP‐VIM (Also measured in MAP, PP)	dementia risk; incidence of cognitive decline	by DSM‐IV, MMSE; 1. all‐cause dementia: DSM‐IV(+), 2. cognitive performance: total points in MMSE	age, sex, educational level, SBP/DBP/MAP/PP, antihypertensive drug use, CAD, type 2 DM, chronic heart failure, AF, TIA or stroke, smoking and dyslipidemia at baseline	SBP‐CV & DBP‐CV positively associated with dementia risk
Wijsman 2016^23^	PROSPER	elderly people with preexisting CVD or risk factors thereof	5606	3‐4 yr	office BP; baseline∼ definite/suspected death from CHD, nonfatal MI, fatal/ nonfatal stroke (median 3.2 yr), per 3mon	75.45 (3.36)	43.2	HTN (%) 84.7 BMI (kg/m2) 27.53 Current smoker (%) 14.2 DM (%) 9.8 Total chol. (mmol/l) 5.72 History of stroke/TIA (%) 10.9 History of MI (%) 15.1 History of vascular disease (%) 46.9	SBP‐SD, DBP‐SD	Incidence of cognitive decline	by Letter‐Digit Coding test, etc.; cognitive decline: pts on each test between baseline & end of f/u	age, sex, country, study treatment (pravastatin/placebo), BMI, education, LDL, HDL, TG, history of vascular disease, history of HTN, history of DM, current smoking, average BP during follow‐up, eGFR, and number of BP lowering medications	No mediation of BP lowering medication in the association between BPV & incidence of cognitive decline
Yamaguchi 2014^33^	N/A (original prospective cohort)	community‐based elderly Japanese	210	4 yr	ambulatory BP; baseline∼ 1day, daytime per 30 min, 14 times, nighttime per 60 min, 6 times	70.9 (0.9)	44.8	HTN (%) 71.9 BMI (kg/m2) 24.2 Current or past smoker (%) 29.5 Current or past drinker (%) 24.8 HbA1c (NGSP) (%) 5.7 Hyperlipidemia (%) 44.3 Total chol. (mg/dl) 203	SBP‐CV, SBP‐SD, SBP‐ARV, DBP‐CV, DBP‐SD, DBP‐ARV	Incidence of cognitive decline	by MMSE; cognitive decline, baseline & 4 yr: > = 1pt MMSE decrease, baseline & 4 yr	age, sex, mean SBP, conventional risk factors (HTN, DM or IGT or IFG, and hyperlipidemia), carotid artery plaque score, and Fazekas grade	SBP‐ARV positively associated with incidence of cognitive decline (no association between DBP‐CV, DBP‐SD, DBP‐ARV regarding incidence of cognitive decline)
Yano 2018^18^	ARIC Study	black and white adults, aged 45 to 64 years	11408	13‐15 yr	office BP; baseline‐2 yr, 3–5 yr, 6–8 yr, 9–11 yr	54.3 (5.7)	44	BMI (kg/m2) 27.5 Current smoker (%) 21 Current drinker (%) 59 DM (%) 7 Total chol. (mg/dl) 214.5″	SBP‐SD, SBP‐VIM, SBP‐ARV, DBP‐SD, DBP‐VIM, DBP‐ARV	Incidence of cognitive decline	by non‐global cognitive test (eg, DWRT, DSST, WFT); cognitive decline: score difference from 9–11 yr to 13–15 yr	demographic variables (age at baseline, sex, race, education, apolipoprotein E ε4 alleles, and study center), clinical characteristics at the index visit (BMI, smoking, alcohol, total chol., HDL, DM, use of antihypertensive drugs, and prevalent stroke); interval from the index visit to the next, mean SBP/DBP, and interactions between BPV parameters and interval	No association between SBPV or DBPV and incidence of cognitive decline
Yoo 2020^30^	KNHIS	adults aged 40 or older	7844814	4‐7 yr	office BP; baseline∼4‐7 yr, per 2 yr (range 3–5 times)	55.5 (10.2)	52.5	HTN (%) 32.8 BMI (kg/m2) 24.2 Current or past smoker (%) 19.5 Current or past drinker (%) 41.8 DM (%) 12.5 Dyslipidemia (%) 16.3 Total chol. (mg/dl) 203.8	SBP‐CV, SBP‐SD, SBP‐VIM, DBP‐CV, DBP‐SD, DBP‐VIM	dementia risk	by ICD; all‐cause dementia/AD/VaD: ICD‐10(+) with the prescription of dementia medication > = 2 times	age, sex, BMI, smoking, alcohol consumption, regular exercise, income status, DM, dyslipidemia, mean SBP/DBP level at baseline, use of antihypertensive drugs, ischemic heart disease, and stroke	SBP‐CV & SBP‐SD & DBP‐CV & DBP‐SD positively associated with dementia risk, including all‐cause dementia, AD, and VaD; SBP‐VIM & DBP‐VIM positively associated with all‐cause dementia risk only

*Abbreviations*: BP, blood pressure; SBP, systolic blood pressure; DBP, diastolic blood pressure; MAP, mean arterial pressure; PP, pulse pressure; SD (in SBP‐SD or DBP‐SD), standard deviation; CV (in SBP‐CV or DBP‐CV), coefficient of variation; VIM (in SBP‐VIM or DBP‐VIM), variance independent of the mean; ARV (in SBP‐ARV or DBP‐ARV), average real variability; BPV, blood pressure variability, SBPV: systolic blood pressure variability; DBPV, diastolic blood pressure variability; DSM, The Diagnostic and Statistical Manual of Mental Disorders; NINCDS‐ADRDA, National Institute of Neurological and Communicative Diseases and Stroke/Alzheimer's Disease and Related Disorders Association; NINDS‐AIREN, International Workshop of the National Institute of Neurological Disorders and Stroke and the Association Internationale pour la Recherche et l'Enseignement en Neurosciences; ADRC, Alzheimer's Disease Research Center; NACC, National Alzheimer's Coordinating Center; MMSE, Mini‐Mental State Examination; 3MSE, Modified Mini‐Mental State Examination; ADAS‐cog, Alzheimer's Disease Assessment Scale–Cognitive Subscale; CDR, Clinical Dementia Rating Sum of Boxes; MoCA, Montreal Cognitive Assessment; CAMCOG, Cambridge Cognitive Examination; DWRT, delayed Word Recall Test; DSST, Digit Symbol Substitution Test; WFT, Word Fluency Test; HTN, hypertension; BMI, body mass index; chol., cholesterol, LDL, low‐density lipoprotein; HDL, high‐density lipoprotein, TG, triglyceride; DM, diabetes mellitus; IGT, impaired glucose tolerance; IFG, impaired fasting glucose; CVD, cardiovascular diseases; AF, atrial fibrillation; MI, myocardial infarction; CABG, coronary artery bypass graft; PAD, peripheral artery disease; CAD, coronary artery disease; TIA, transient ischemic attack; CIV, cerebral infarct volume; WMH, white matter hyperintensities; ICH, intracerebral hemorrhage; NIHSS, National Institute of Health Stroke Scale; MCI, mild cognitive impairment; AD, Alzheimer's disease; CHD, coronary heart disease; ECG, electrocardiography; VaD, vascular dementia; HbA1c, Hemoglobin A1c; NPSG, National Patient Safety Goals; eGFR, estimated glomerular filtration rate; MDRD, Modification of Diet in Renal Disease; ICD, International Classification of Disease; ONTARGET, Ongoing Telmisartan Alone and in Combination with Ramipril Global End point Trial; TRANSCEND, Telmisartan Randomized Assessment Study in ACE Intolerant Subjects with Cardiovascular Disease; ADNI, Alzheimer's Disease Neuroimaging Initiative; PSCI, Post‐stroke cognitive impairment; WHIMS‐MRI, Women's Health Initiative Memory MRI study; preDIVA trial, Prevention of Dementia by Intensive Vascular Care trial; S.AGES, Sujets AGÉS‐ Aged Subjects; PROSPER, PROspective Study of Provastatin in the Elderly at Risk; ARIC Study, Atherosclerosis Risk in Communities Study; KNHIS, Korean National Health Insurance Service; N/A, not applicable; pt, point; ave, average; f/u, follow‐up; wk, week; yr, year; and mon, month.

^a^Classified as office blood pressure (BP), home BP, or ambulatory BP.

^b^Expressed as mean (SD) generally.

^c^Including HTN, obesity, smoking, alcohol use, DM, cholesterol, CVD, depression, etc.

^d^In terms of cognitive decline, global scales would be mentioned as priority.

^e^Positive results were shown mainly. Additional notes for the findings regarding DBPV would be put in parentheses.

^f^Significant potential confounders derived from univariate analyses.

^g^Including MI, CABG, angioplasty, stroke, and PAD.

^h^SDreg indicates SD about participant's regression line.

^i^RMSE, abbreviated from root‐mean‐square error, is calculated from the linear regression of BP readings on the participant's age (yr) at BP measurement.

### 2.6 Assessment of the risk of bias

Risk of bias was independently assessed by the two reviewers according to the Quality in Prognostic Factor Studies (QUIPS).^19^ Disagreements were jointly reassessed to reach a final consensus.

### 2.7 Data synthesis and analysis

Studies with sufficient quantitative results were further analyzed by two‐step meta‐analysis. First, a traditional meta‐analysis was employed to integrate the most‐adjusted hazard ratio (HR) or odds ratio (OR) in each outcome from the highest versus lowest BPV groups of different BPV indices across different studies, using RevMan 5.4 (Cochrane, UK). For groups showing significant associations, a further dose‐response meta‐analysis was conducted using the *dosresmeta* R package with a linear model.^20^ In the dose‐response meta‐analysis, the hazard ratio was approximated as the relative risk.^20^ Subgroup analysis was carried out between BPV and outcome of interests according to the BPV timeframes, according to different follow‐up durations for cognitive performance, and according to the mean age of participants in included studies with the cutoff age at 65 years old, using RevMan 5.4 (Cochrane, UK). Sensitivity analysis was also conducted between BPV and outcome of interests using the leave‐one‐out meta‐analysis function from the *meta* R package. Subgroup analysis and sensitivity analysis were performed when the meta‐analysis contained more than two studies. The traditional meta‐analysis and the leave‐one‐out meta‐analysis were conducted using the inverse variance method and the random effects model.

The heterogeneity among included studies was evaluated by the Cochrane Q‐test, with a significance level of 0.05, and I^2^ statistics, where an I^2^ > 60%, was considered highly heterogenous.^21^ Publication bias was assessed by producing a funnel plot and conducting the Egger's test to examine the symmetry of the funnel plot, using the *metafor* R package, for outcomes integrated from more than two studies.^22^

## 3 RESULTS

### 3.1 Study selection & study characteristics

**FIGURE 1:**
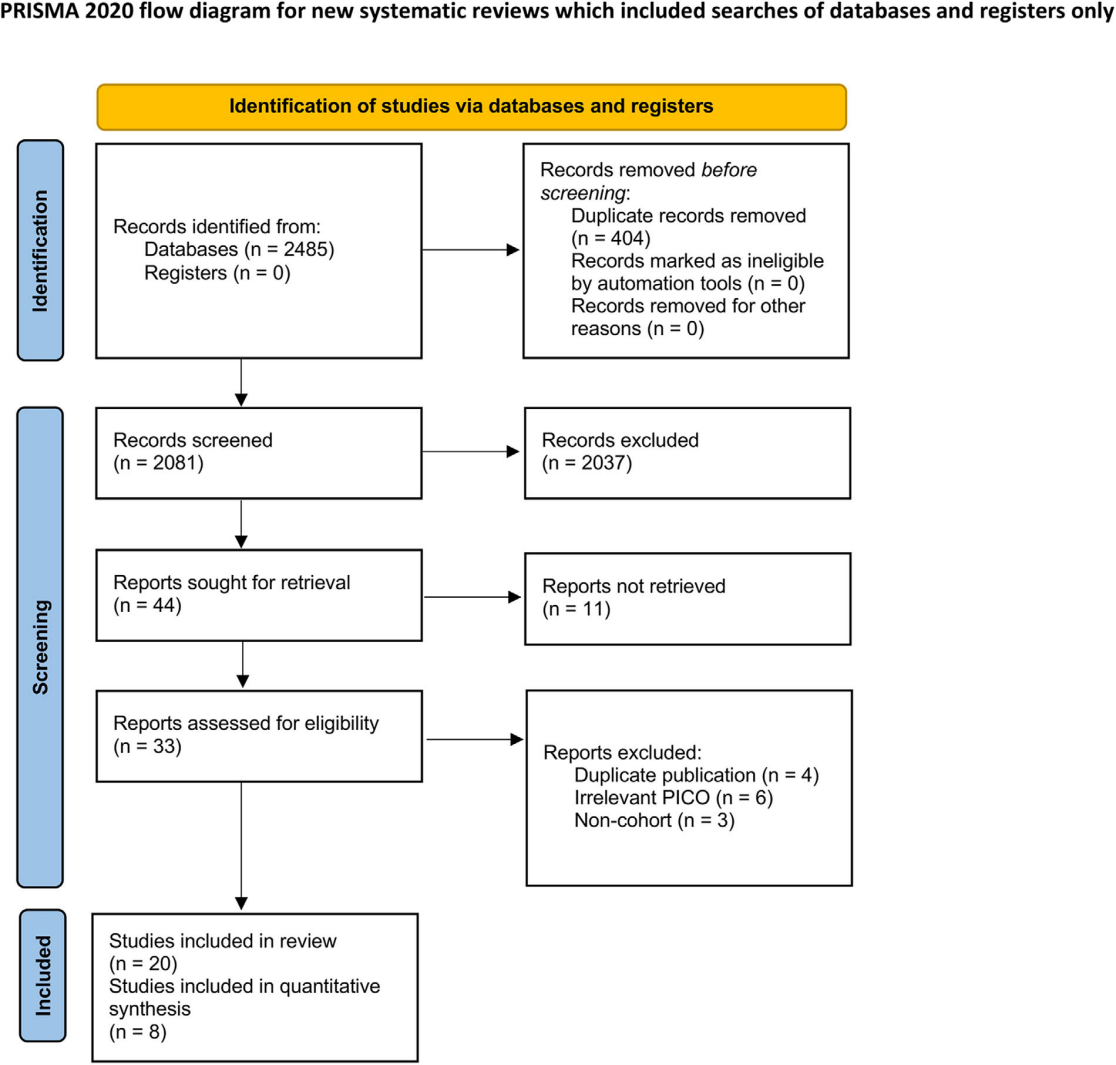
Study flowchart. PICO indicates the acronym from patient, intervention, comparison, and outcome

We obtained 2485 records in total on our database search. Using the PRISMA flowchart (Figure 1), 20 studies were included for qualitative analysis, among which eight studies were included in the primary meta‐analysis, and three in the further dose‐response analysis.

The characteristics of included studies are shown in Table 1. In short, 7 924 168 individuals over a follow‐up duration of 3 months to 22 years (mean age range, 54.3–84.4 years; 52.4% male) were included. Some studies required participants with specific comorbidities such as stroke or cardiovascular disease.^23^, ^24^, ^25^, ^26^, ^27^, ^28^ The remaining studies included the general population, and two studies by Haring (2019)^29^ and Liu (2015)^30^ excluded individuals with comorbidities, including diabetes mellitus, coronary heart disease, and/or stroke. Among BPV measurement modalities, office BP was adopted in 15 (75%) studies, home BP in 4 (20%) studies, and ambulatory BP in 2 (10%) studies. For BPV timeframes, 13 (65%) studies measured long‐term BPV, 3 (15%) studies mid‐term BPV, and 4 (20%) studies short‐term. For BPV indices, CV was the most frequently used one for both SBPV (15 studies; 75%) and DBPV (12 studies; 60%). The most commonly used cognitive test was the MMSE, which was reported in 10 out of 16 studies (63%).

### 3.2 Assessment of risk of bias

**FIGURE 2:**
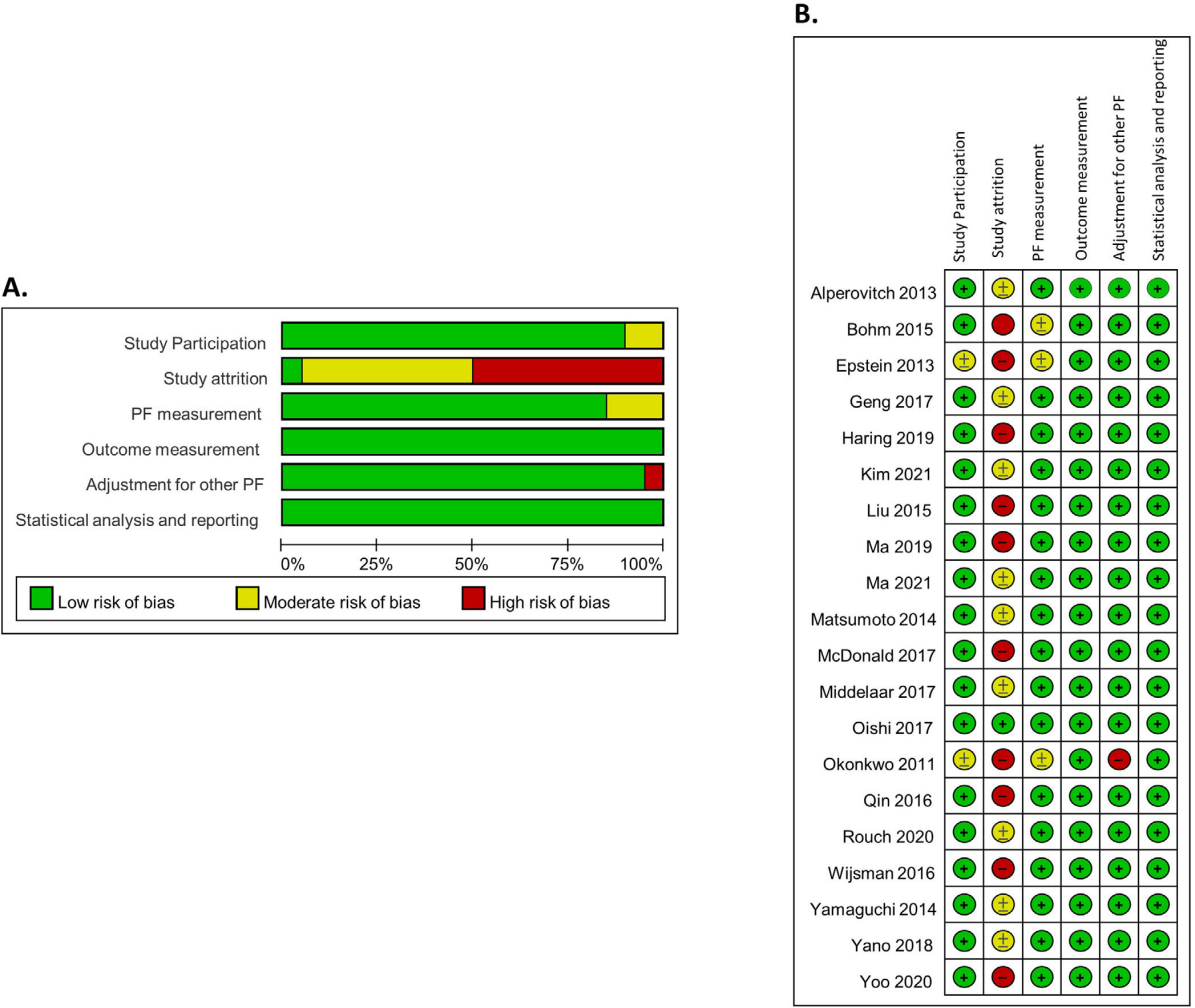
Risk of bias (ROB) assessment in graph (panel A) and summary (panel B). The ROB of 20 included studies was evaluated with the Quality in Prognostic Factor Studies (QUIPS) tool, which included six domains, with each domain comprising several signaling items. PF indicates prognostic factors

The risk of bias was assessed with the QUIPS tool (Figure 2A and 2B). Except for a moderate to high risk for “study attrition” due to the insufficient description of reasons for loss to follow‐up and the characteristics of participants lost to follow‐up, nearly all studies were generally rated at low risk of bias in all items in the QUIPS tool.

### 3.3 Association between BPV indices and dementia risk

The relationships between BPV indices and dementia risk are summarized in the upper part of Table 2, and forest plots for each meta‐analysis are shown in Figure S1‐S6. All studies were measured using either mid‐term or long‐term BPV. SBP‐CV and SBP‐SD showed significant positive correlations with all‐cause dementia risk (SBP‐CV: HR = 1.45 [95% CI, 1.11–1.90] I^2 ^= 78%; SBP‐SD: HR = 1.31 [95% CI, 1.03–1.67] I^2 ^= 70%), whereas SBP‐VIM and DBP‐CV demonstrated no significant association. Alzheimer's disease and vascular dementia had no significant associations with any analyzed BPV index. All primary results for HRs of dementia risk had high heterogeneities (I^2 ^= 66%‐90%), except for the analysis on SBP‐VIM versus vascular dementia risk (I^2 ^= 16%). Subgroup analysis according to different BPV timeframes (Figure S8‐S10) yielded consistent associations between SBP‐CV or SBP‐SD and all‐cause dementia risk, with a more obvious effect of mid‐term SBPV over long‐term one, as well as an additional significant positive association between mid‐term DBP‐CV and all‐cause dementia risk. Subgroup analysis according to different outcome follow‐up durations (Figure S11 and S12) showed a similar trend between SBP‐CV or SBP‐SD and all‐cause dementia risk, with a more apparent effect on “5 to 10 years” follow‐up over “more than 10 years” one. Subgroup analysis according to the mean age of participants in included studies (Figure S13‐15) showed similar findings between SBP‐CV, SBP‐SD, or DBP‐CV and all‐cause dementia risk, with a possible trend of higher dementia risk among the elderly subgroup. Sensitivity analysis by leave‐one‐out meta‐analysis (Figure S16‐S18) was conducted for meta‐analysis with more than three studies. The pooled results after omitting one study at a time remained robust for the association between SBP‐CV and all‐cause dementia risk (Figure S16). However, after omitting one study, some of the pooled results became insignificant for the SBP‐SD (Figure S17) and DBP‐CV (Figure S18).

**TABLE 2:** Summarized results of the associations of different BPV indices with different phenotypes of dementia risk or incidence of cognitive decline

All‐cause dementia					
HR [95%CI], (n, p, I^2^)	**CV**	**SD**	VIM	ARV	Full range*
SBP	**1.45 [1.11, 1.90]** **(*n* = 4, *p* = .006, I^2 ^= 78%)**	**1.31 [1.03, 1.67]** **(*n* = 3, *p* = .03, I^2 ^= 70%)**	1.44 [0.87, 2.40] (*n* = 2, *p* = .16, I^2 ^= 82%)	nil	nil
DBP	1.64 [0.96, 2.81] (*n* = 3, *p* = .07, I^2 ^= 87%)	nil	nil	nil	nil
Alzheimer's disease					
HR [95%CI], (n, p, I^2^)	CV	SD	VIM	ARV	Full range*
SBP	1.51 [0.80, 2.86] (*n* = 2, *p* = .20, I^2 ^= 83%)	1.47 [0.81, 2.68] (*n* = 2, *p* = .20, I^2 ^= 77%)	1.46 [0.82, 2.57] (*n* = 2, *p* = .20, I^2 ^= 80%)	nil	nil
DBP	1.71 [0.68, 4.28] (*n* = 2, *p* = .25, I^2 ^= 90%)	nil	nil	nil	nil
Vascular dementia					
HR [95%CI], (n, p, I^2^)	CV	SD	VIM	ARV	Full range*
SBP	1.57 [0.71, 3.46] (*n* = 2, *p* = .27, I^2 ^= 66%)	1.83 [0.59, 5.63] (*n* = 2, *p* = .30, I^2 ^= 71%)	1.24 [0.96, 1.60] (*n* = 2, *p* = .11, I^2 ^= 16%)	nil	nil
DBP	1.78 [.62, 5.11] (*n* = 2, *p* = .29, I^2 ^= 75%)	nil	nil	nil	nil
Cognitive decline					
OR [95%CI], (n, p, I^2^)	CV	SD	VIM	ARV	Full range*
SBP	2.32 [0.67, 8.08] (*n* = 2, *p* = .19, I^2 ^= 87%)	nil	nil	nil	nil
DBP	nil	nil	nil	nil	nil

* Calculated as the difference between the maximum and the minimum.

*Abbreviations*: CV, coefficient of variation; SD, standard deviation; VIM, variance independent of the mean; ARV, average real variability; HR, hazard ratio; OR, odds ratio; SBP, systolic blood pressure; DBP, diastolic blood pressure.

### 3.4 Association between BPV indices and incidence of cognitive decline

Results on the incidence of cognitive decline are shown in the lower part of Table 2 and in Figure S7. There were two studies using short‐term BPV, along with one mid‐term study and one long‐term study. Only the association between SBP‐CV and cognitive decline was available for meta‐analysis, and insignificant results were found, with a high heterogeneity (I^2^ = 87%). All subgroup analysis and sensitivity analysis were not conducted due to the limited study number.

### 3.5 Further dose response between SBP‐CV or SBP‐SD and all‐cause dementia risk

**FIGURE 3:**
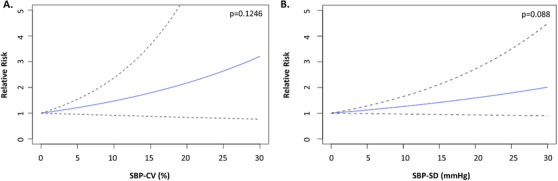
Linear dose‐response relationship between all‐cause dementia risk and coefficient of variation of systolic blood pressure (SBP‐CV, panel A) and standard deviation of systolic blood pressure (SBP‐SD, panel B). The dose‐response analysis was conducted secondly for the pairs of blood pressure variability (BPV) indices and outcomes of interests which showed statistically significant associations firstly in highest‐to‐lowest BPV comparisons. Hazard ratios were approximated as the relative risks. No significant correlations were observed

Further dose response analysis was conducted between the risk of all‐cause dementia and SBP‐CV or SBP‐SD. As shown in Figure 3A (SBP‐CV) and 3B (SBP‐SD), no significance was found using a linear dose‐response model (SBP‐CV: *p* = .1246; SBP‐SD: *p* = .088).

### 3.6 Assessment of publication bias

The funnel plots and subsequent Egger tests were only conducted for those groups with > 2 studies (Figure S19). The results showed no evidence of publication bias.

## 4 DISCUSSION

This study included 20 cohort studies with a total of 7 924 168 participants; we conducted a traditional meta‐analysis from eight and further dose‐response analysis from three included studies. As shown in the Table 1, the data extracted were from the fully‐adjusted models, which took common confounders such as baseline BP, sex, and age into consideration. Nevertheless, subgroup analysis was conducted according to the mean age due to the heterogeneity of the included study population with a wide age range.

We identified that higher SBPV, particularly SBP‐CV and SBP‐SD, was potentially associated with a higher risk of dementia with HRs around 1.3–1.4. In the subgroup analysis according to different BPV timeframes, different outcome follow‐up durations, and the mean age of participants in included studies, a consistent trend was found for the associations between BPV and dementia risk, which is more prominent in groups with shorter BPV timeframes, shorter follow‐up durations, and an older age. Sensitivity analysis remained robust for meta‐analysis with sufficient included studies of more than three. The dose‐response meta‐analysis demonstrated no significant dose‐response between SBPV and all‐cause dementia risk. The heterogeneity is high in most of our analyses.

### 4.1 Prognostic value of SBPV or DBPV for cognitive dysfunction

Higher SBPV was significantly associated with higher all‐cause dementia risk but was not specifically associated with the dementia subtypes included in our study, Alzheimer's disease and vascular dementia. In addition, the association between SBPV and dementia risk was more prominent in subgroups with shorter follow‐up duration for cognitive performances and with older ages. The possible mechanism accounting for this finding remains to be elucidated. For the other outcome of interest, the incidence of cognitive decline, was not analyzed for almost all BPVs due to limited data report.

For DBPV, in some of the included studies, the effect estimates of DBPV were not provided because of the statistically insignificant results on cognitive dysfunction (Table 1); therefore, the results of DBPV in our meta‐analysis should be interpreted with caution.

Our systematic review represents the summarized totality of evidence by including the most comprehensive and updated studies, as compared with a previous review.^11^ The recent systematic review and meta‐analysis showed a possible relationship between BPV, either SBPV or DBPV, and all‐cause dementia risk in late life,^11^ which was similar to our findings that the elderly with higher BPV bore more dementia risk than the non‐elderly in our subgroup analysis according to the mean age. Both of them were statistically significant and had higher risk ratios than our findings; however, they only included two studies by Alperovitch (2013)^31^ and Oishi (2017).^32^ For the relationship between SBPV and all‐cause dementia risk, we analyzed two more studies by Ma (2019)^33^ and Yoo (2020)^34^; for DBPV and all‐cause dementia risk, we included one more study by Yoo (2020).^34^ These extra studies showed less pronounced effects, which may be accounted for the discrepancy.

### 4.2 Prognostic value of cognitive dysfunction by different BPV indices

Since the SD of BP was related to mean BP, CV and VIM were further utilized for BPV indices to adjust for mean BP.^6^ In our study, only SBP‐CV and SBP‐SD were associated with all‐cause dementia risk. Although no statistical significance was found between SBP‐VIM and all‐cause dementia risk, the individual ORs in both of the two included studies in that meta‐analysis were statistically significant. In comparison with the included study number regarding SBP‐CV and SBP‐SD of four and three respectively, chances are the limited study number resulted in large uncertainty and in turn yielded an insignificant pooled result. CV and SD were the most common BPV indices in long‐term BPV with prognostic relevance for cardiovascular, and subclinical renal impairment.^35^ SBP‐CV and SBP‐SD were also found to be related to cardiovascular events and all‐cause mortality.^36^ Our results extend the impact of SBP‐CV and SBP‐SD from that of recently established clinical relevance to cognitive dysfunction. Furthermore, SBP‐CV was the most reported variability parameter in our systematic review, and had a stronger association with dementia over a follow‐up duration of 4–22 years for cognitive performance compared to SBP‐SD. It is possible that SBP‐CV has a better prognostic value for all‐cause dementia risk.

No association was observed between all DBPV indices and dementia risk of all types or incidence of cognitive decline. The statistically insignificant finding between overall DBPV and dementia risk of all types or incidence of cognitive decline might imply that DBPV plays a less important role in cognitive dysfunction.

### 4.3 Prognostic value of cognitive dysfunction by different BPV timeframes

In both SBP and DBP, the variability derived from a shorter timeframe was associated with stronger associations with all‐cause dementia risk in this study. This might shed some light onto the different clinical correlation of BPV estimated with different timeframes.^35^ Possible proposed mechanism would be discussed below.

### 4.4 Heterogeneity and publication bias of the included studies

Most of the data in the included studies showed statistically significant trends between BPV and cognitive dysfunction; however, in our meta‐analysis, the high heterogeneity across those studies gave rise to statistically insignificant pooled effect estimates in most of our analyses (Table 2). This heterogeneity might result from diverse study characteristics (Table 1). Given the inevitable differences in designs and participant characteristics, more studies are needed.

### 4.5 Proposed mechanism underlying dementia based on SBPV

BPV could be considered as a comprehensive reflection of the interaction between cardiovascular physiological responses (eg, neurohumoral reflex) and environmental settings (eg, arterial elastic properties and seasonal changes), although the exact details are not yet completely understood. Certain vascular diseases (eg, stroke and cerebral small‐vessel disease) were found to be associated with SBPV,^9^ and the effect of SBPV on the brain parenchyma or vasculature could be visualized on neuroimaging studies.^29^, ^37^ Compared with SBPV, the role of DBPV in the pathophysiological process of cognitive dysfunction is debatable.^29^, ^37^ Therefore, high SBPV, rather than DBPV, could be considered a manifestation of hemodynamic dysregulation, which may corroborate our results that SBPV is a better indicator for dementia. The proposed vascular pathophysiology through which cognitive dysfunction developed were manifested by the association of BPV and all‐cause dementia risk, specifically vascular dementia,^34^, ^38^, ^39^, ^40^ yet the associations with all individual dementia subtypes were insignificant in our study. The inconsistent findings might stem from the limited study numbers. Similar findings were also seen in our sensitivity analysis by omitting one study at a time, with robustness preserved only in analysis with four and above included studies. As for the timeframes from which BPV was derived, mid‐term BPV showed stronger associations with dementia risk than long‐term one. Previous studies showed the effect of mid‐term BPV elevation on aortic stiffness increase and carotid arterial remodeling maladaptation, post‐stroke cognitive decline, and all‐cause death, which might also be explained by neurohormonal hemodynamic dysregulation proposed as above, yet limited evidence compare the effect of mid‐term with long‐term BPV.^8^, ^41^, ^42^ For different cognitive follow‐ups, the effect of BPV was more prominent on shorter follow‐ups, which might imply elevated BPV as an indicator of earlier disease onset with higher risk of total disease incidence. More studies were warranted to investigate this effect. As observed in our study, BPV was more prognostic for the elderly aged 65 years and above. Our findings agreed well with the findings of previous studies.^43^, ^44^, ^45^

### 4.6 Strengths and limitations

The strengths of our study are, first, this is the largest meta‐analysis of cohort studies providing insights into the prognostic value of BPV in cognition. Second, a wide range of different BPV indices was studied from the perspective of different BPV statistical indices comprising overall variability, variability between consecutive visits, and extreme values on a single visit, and a broad‐spectrum of BPV timeframes ranging from short‐term, mid‐term, to long‐term ones. Third, dementia risk and incidence of cognitive decline, the outcomes of interest in our study, were of clinical importance, since they posed a significant threat to care burden.^46^

However, there were some limitations in this study for the interpretation of results. First, because of the different characteristics of the study populations included in this meta‐analysis, the comorbidity composition difference between the included studies may be a significant source of heterogeneity (Table 1). The quality of evidence was inevitably influenced by the high heterogeneity and limited number of studies, as shown in our sensitivity analysis. However, subgroup analysis or meta‐regression based on the possible confounders mentioned above was not possible in our meta‐analysis because of the limited number of studies. To be noted, the fact that all‐cause dementia risk was related to SD and CV but not VIM might suggest that the BP level may still have a greater influence than true variability. Second, most of the BPV measured in this meta‐analysis was categorized as long‐term. Investigations covering other types of BPV, such as very short‐term or short‐term, may be a direction for future investigation. Third, difficulties in data synthesis result from the highly variable presentations of the measures for BPV and cognitive dysfunction of these included studies. Therefore, we adopted the most available outcome measure, the effect estimates between the highest and lowest BPV groups, which is in line with that of a previous systematic review.^11^ Fourth, lack of availability of data reports, particularly the effect estimates of insignificant results in individual studies, resulted in insufficient primary analysis and sensitivity analysis in our review.

## CONFLICT OF INTEREST

None.

## AUTHOR CONTRIBUTIONS

**Tzu‐Jung Chiu**: Conception and design of the study, acquisition and analysis of the data, drafting and revising the manuscript. **Jiunn‐Tyng Yeh**: Conception and design of the study, acquisition and analysis of the data, drafting and revising the manuscript. **Chi‐Jung Huang**: Conception and design of the study, analysis and interpretation of the data, drafting and revising the manuscript. **Chern‐En Chiang**: Conception of the study, analysis and interpretation of the data, revising the manuscript. **Shih‐Hsien Sung**: Conception of the study, analysis and interpretation of the data, revising the manuscript. **Chen‐Huan Chen**: Conception of the study, analysis and interpretation of the data, revising the manuscript. **Hao‐Min Cheng**: Conception and design of the study, acquisition, analysis and interpretation of the data, drafting and revising the manuscript. All the author approved the final version of the manuscript and agreed to be accountable for all aspects of the work in ensuring that questions related to the accuracy or integrity of any part of the work are appropriately investigated and resolved.

## Supporting information

Supporting information
