# Supplementary material for: Blood pressure variability and cognitive dysfunction: A systematic review and meta‐analysis of longitudinal cohort studies
Source: J Clin Hypertens (Greenwich). 2021 Jun 21;23(8):1463–82. doi: 10.1111/jch.14310 (PMC8678719; doi:10.1111/jch.14310)
Supplement: Supplementary file 1 — Supporting information [file JCH-23-1463-s001.doc]

**Figure S1. Association of all-cause dementia risk with different indices of SBPV**

**
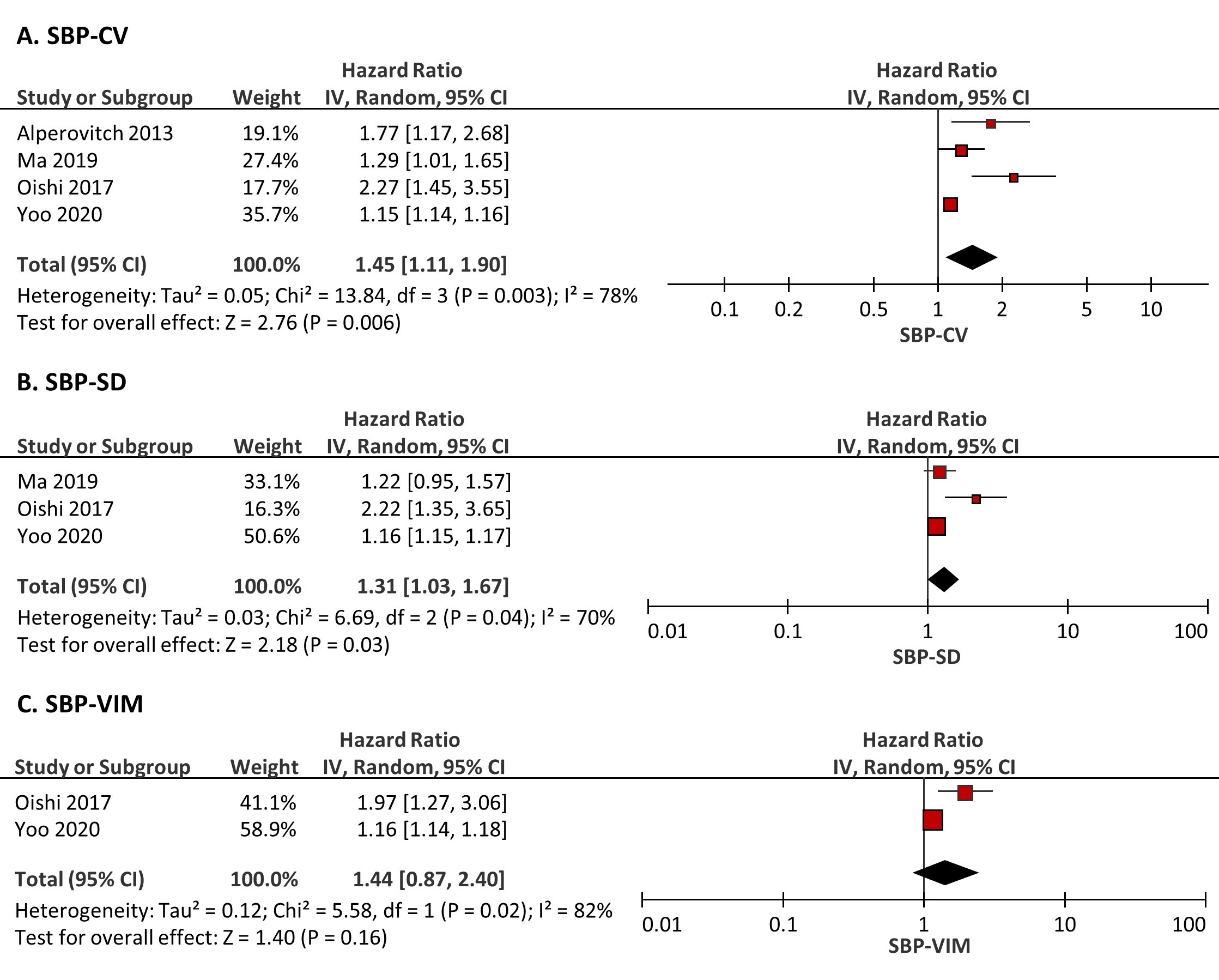
**

SBP indicates systolic blood pressure; SBPV, variability of systolic blood pressure; CV, coefficient of variation; SD, standard deviation; VIM, variance independent of the mean; and IV, inverse variance. SBP-CV indicates CV of SBP; similar expressions apply to SBP-SD and SBP-VIM. The forest plots were produced with RevMan 5.4 (Cochrane, UK) using the inverse variance method with random effects.

**Figure S2. Association of all-cause dementia risk with different indices of DBPV**


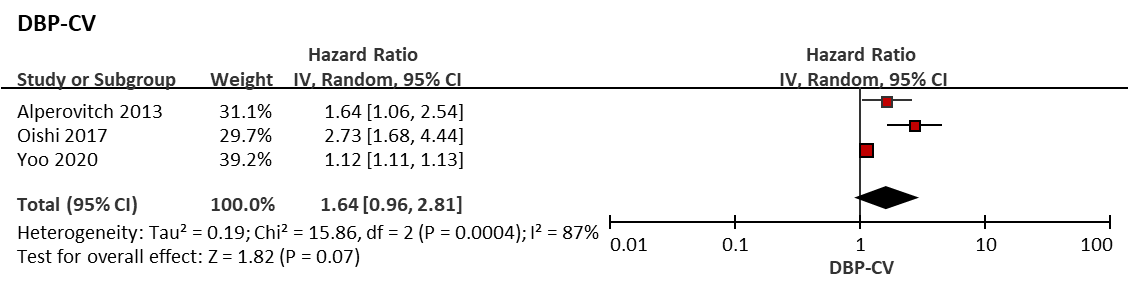


DBP indicates diastolic blood pressure; DBPV, variability of diastolic blood pressure; CV, coefficient of variation; and IV, inverse variance. DBP-CV indicates CV of DBP. The forest plot was produced with RevMan 5.4 (Cochrane, UK) using the inverse variance method with random effects.

**Figure S3. Association of Alzheimer’s disease risk with different indices of SBPV**

**
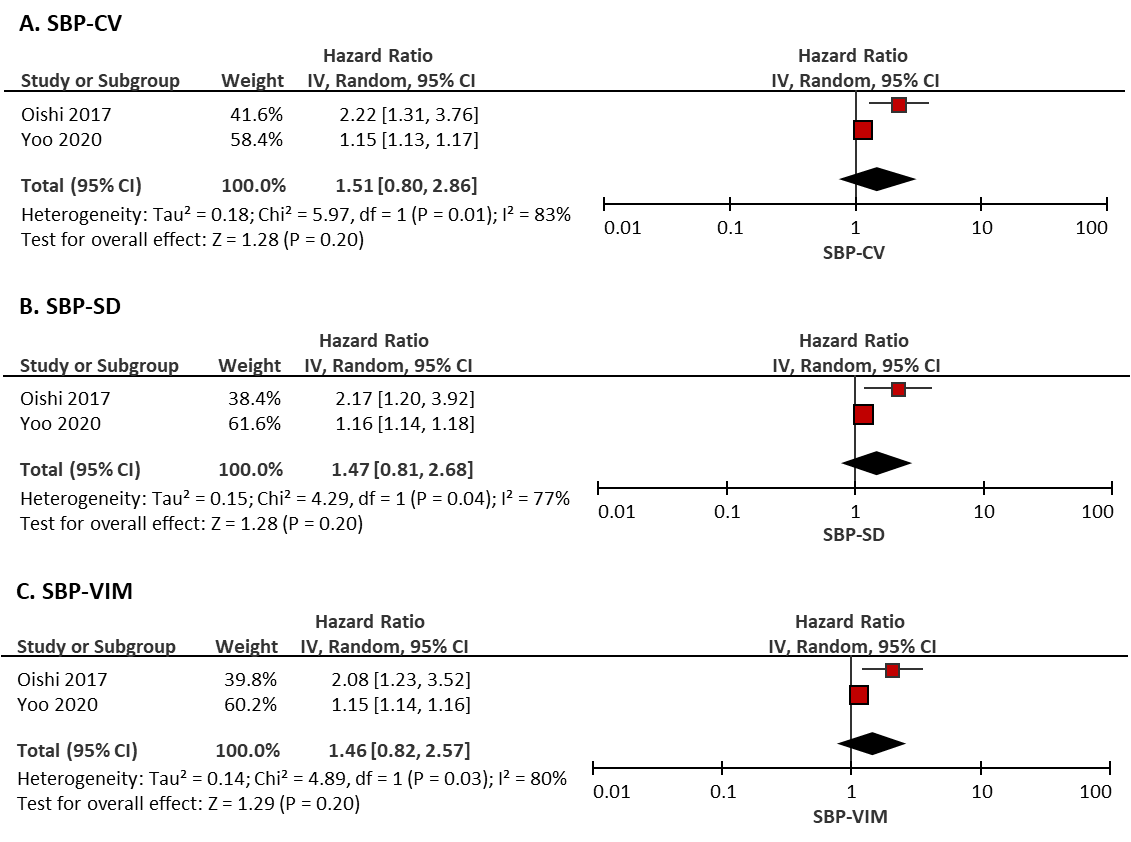
**

SBP indicates systolic blood pressure; SBPV, variability of systolic blood pressure; CV, coefficient of variation; SD, standard deviation; VIM, variance independent of the mean; and IV, inverse variance. SBP-CV indicates CV of SBP; similar expressions apply to SBP-SD and SBP-VIM. The forest plots were produced with RevMan 5.4 (Cochrane, UK) using the inverse variance method with random effects.

**Figure S4. Association of Alzheimer’s disease risk with different indices of DBPV**

**
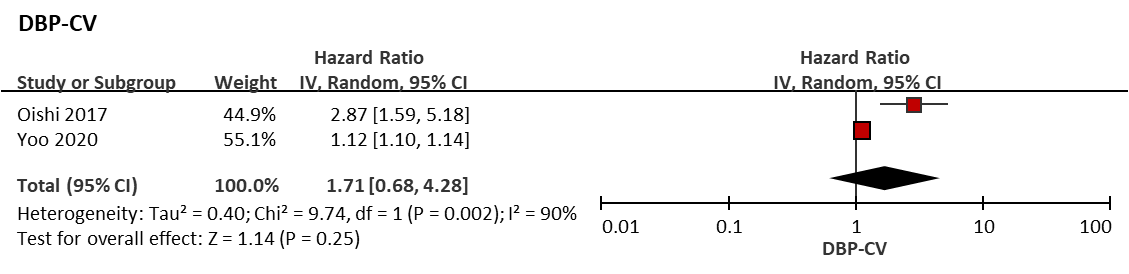
**

DBP indicates diastolic blood pressure; DBPV, variability of diastolic blood pressure; CV, coefficient of variation; and IV, inverse variance. DBP-CV indicates CV of DBP. The forest plot was produced with RevMan 5.4 (Cochrane, UK) using the inverse variance method with random effects.

**Figure S5. Association of vascular dementia risk with different indices of SBPV**

**
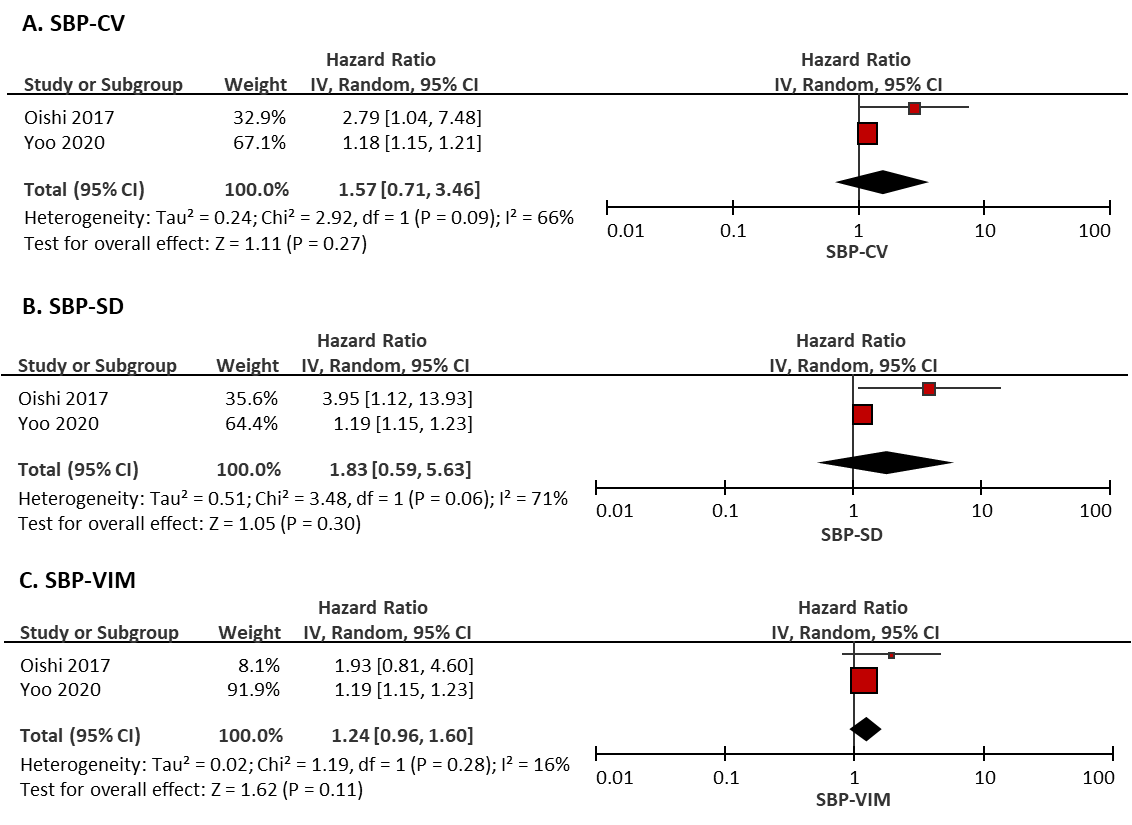
**

SBP indicates systolic blood pressure; SBPV, variability of systolic blood pressure; CV, coefficient of variation; SD, standard deviation; VIM, variance independent of the mean; and IV, inverse variance. SBP-CV indicates CV of SBP; similar expressions apply to SBP-SD and SBP-VIM. The forest plots were produced with RevMan 5.4 (Cochrane, UK) using the inverse variance method with random effects.

**Figure S6. Association of vascular dementia risk with different indices of DBPV**

**
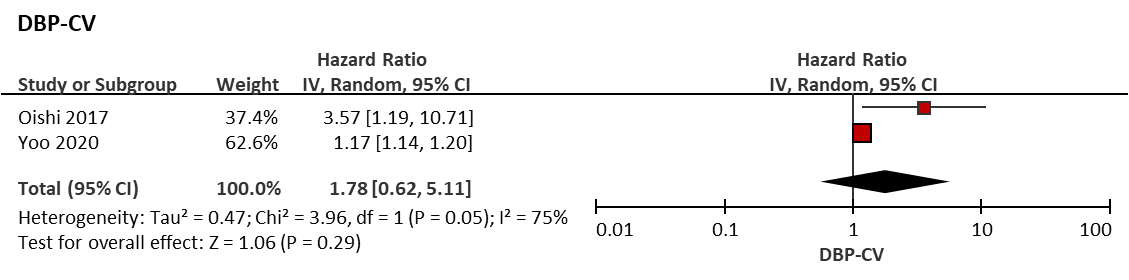
**

DBP indicates diastolic blood pressure; DBPV, variability of diastolic blood pressure; CV, coefficient of variation; and IV, inverse variance. DBP-CV indicates CV of DBP. The forest plot was produced with RevMan 5.4 (Cochrane, UK) using the inverse variance method with random effects.

**Figure S7. Association of incidence of cognitive decline with different indices of SBPV**

**
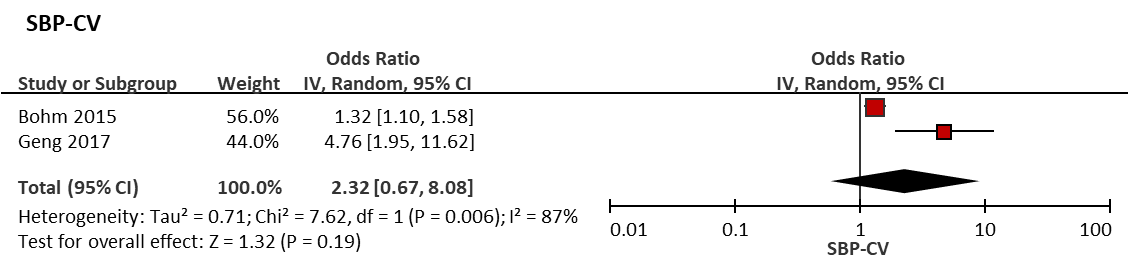
**

SBP indicates systolic blood pressure; SBPV, variability of systolic blood pressure; CV, coefficient of variation; and IV, inverse variance. SBP-CV indicates CV of SBP. The forest plot was produced with RevMan 5.4 (Cochrane, UK) using the inverse variance method with random effects.

**Figure S8. Subgroup analysis between SBP-CV and all-cause dementia risk according to different timeframes of BPV**

**
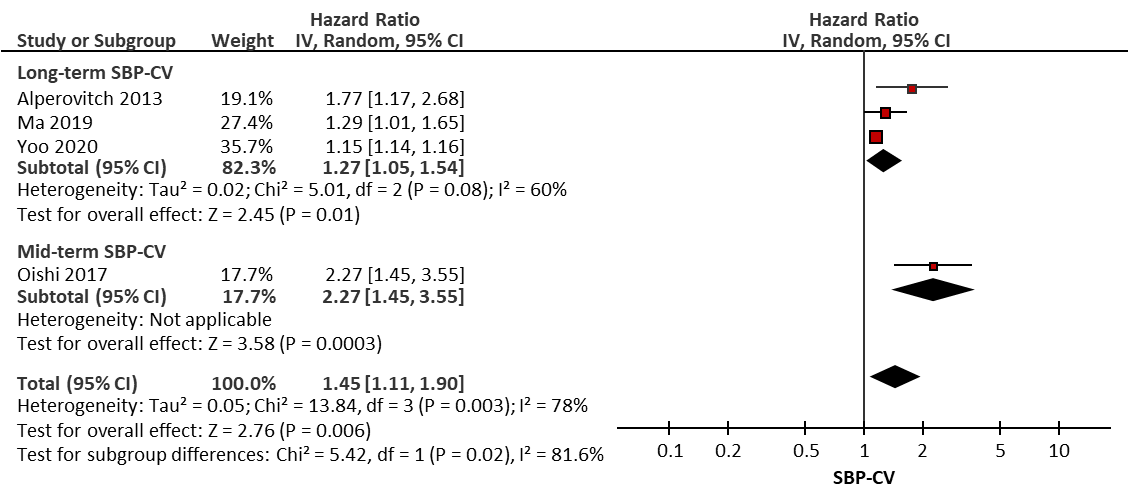
**

SBP indicates systolic blood pressure; BPV, variability of blood pressure; CV, coefficient of variation; and IV, inverse variance. SBP-CV indicates CV of SBP. The forest plot was produced with RevMan 5.4 (Cochrane, UK) using the inverse variance method with random effects.

**Figure S9. Subgroup analysis between SBP-SD and all-cause dementia risk according to different timeframes of BPV**


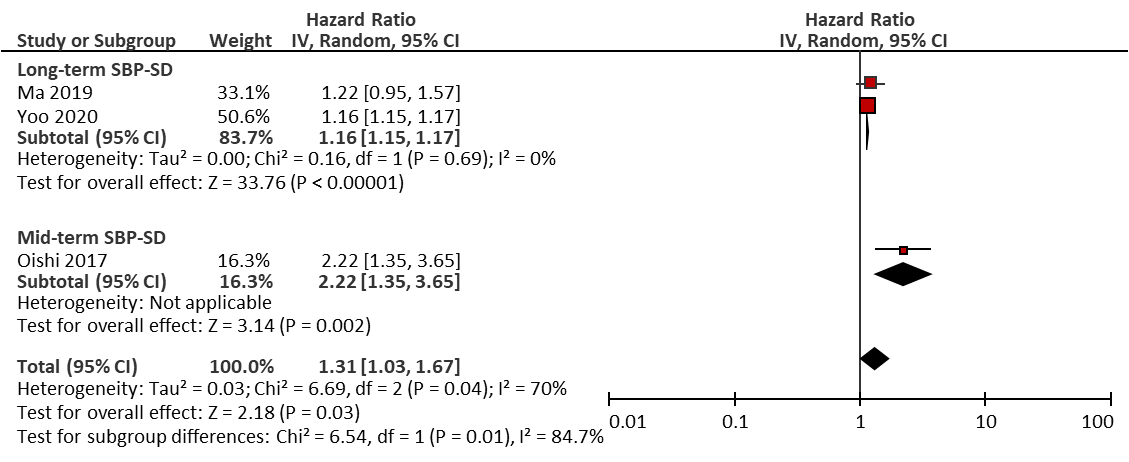


SBP indicates systolic blood pressure; BPV, variability of blood pressure; SD, standard deviation; and IV, inverse variance. SBP-SD indicates SD of SBP. The forest plot was produced with RevMan 5.4 (Cochrane, UK) using the inverse variance method with random effects.

**Figure S10. Subgroup analysis between DBP-CV and all-cause dementia risk according to different timeframes of BPV**

**
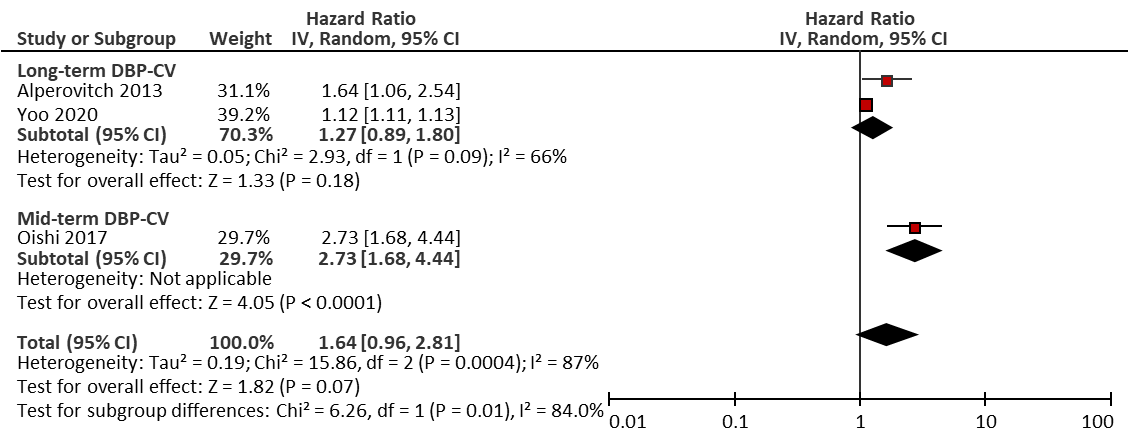
**

DBP indicates diastolic blood pressure; BPV, variability of blood pressure; CV, coefficient of variation; and IV, inverse variance. DBP-CV indicates CV of DBP. The forest plot was produced with RevMan 5.4 (Cochrane, UK) using the inverse variance method with random effects.

**Figure S11. Subgroup analysis between SBP-CV and all-cause dementia risk according to different follow-up durations for cognitive performances**

**
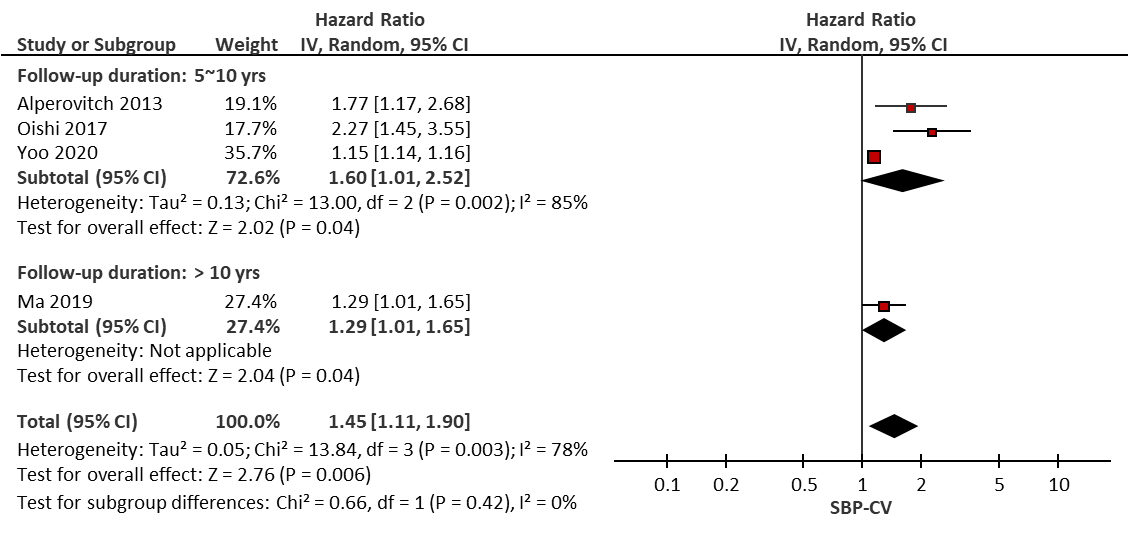
**

SBP indicates systolic blood pressure; CV, coefficient of variation; and IV, inverse variance. SBP-CV indicates CV of SBP. The forest plot was produced with RevMan 5.4 (Cochrane, UK) using the inverse variance method with random effects.

**Figure S12. Subgroup analysis between SBP-SD and all-cause dementia risk according to different follow-up durations for cognitive performances**

**
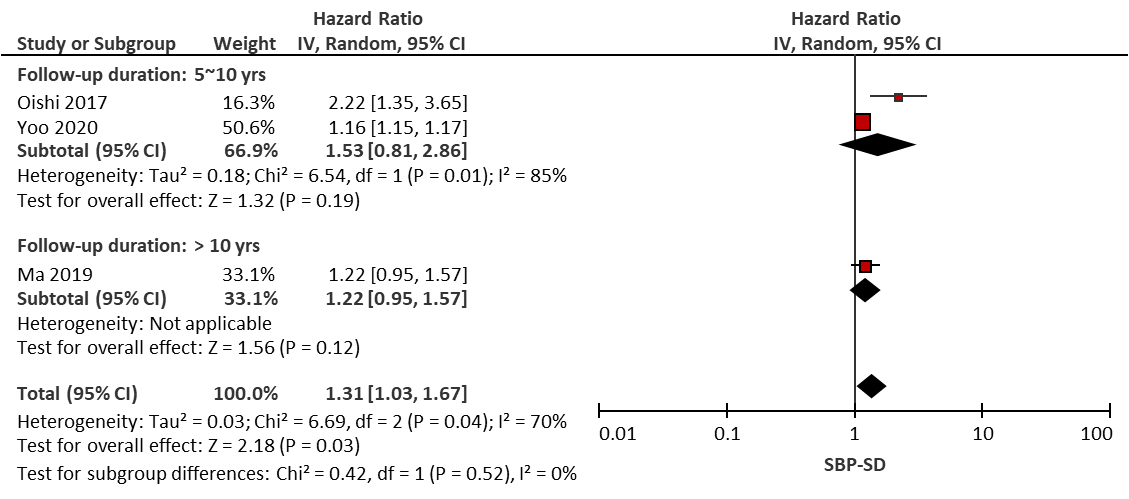
**

SBP indicates systolic blood pressure; SD, standard deviation; and IV, inverse variance. SBP-SD indicates SD of SBP. The forest plot was produced with RevMan 5.4 (Cochrane, UK) using the inverse variance method with random effects.

**Figure S13. Subgroup analysis between SBP-CV and all-cause dementia risk according to the mean age of participants in included studies**

**
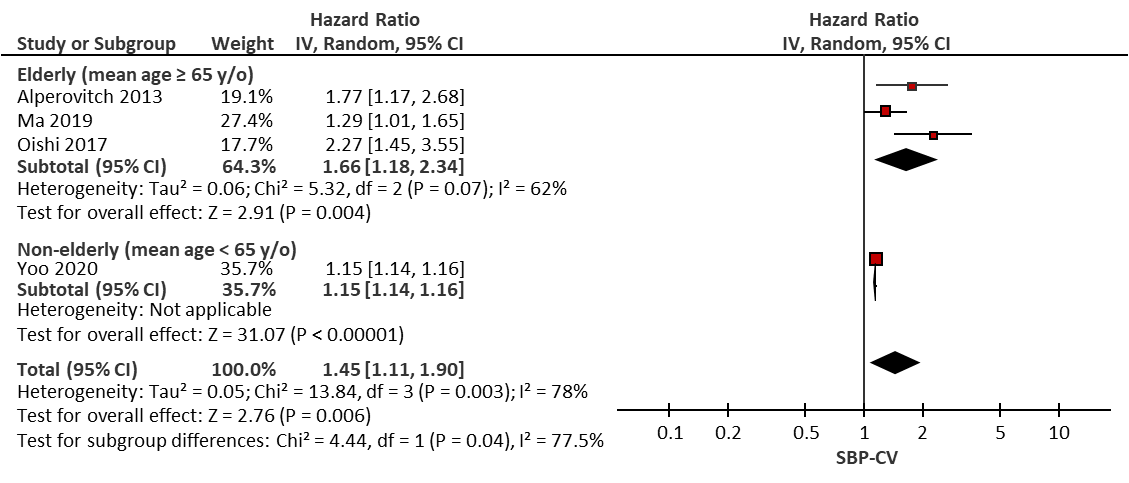
**

SBP indicates systolic blood pressure; CV, coefficient of variation; and IV, inverse variance. SBP-CV indicates CV of SBP. The forest plot was produced with RevMan 5.4 (Cochrane, UK) using the inverse variance method with random effects.

**Figure S14. Subgroup analysis between SBP-SD and all-cause dementia risk according to the mean age of participants in included studies**

**
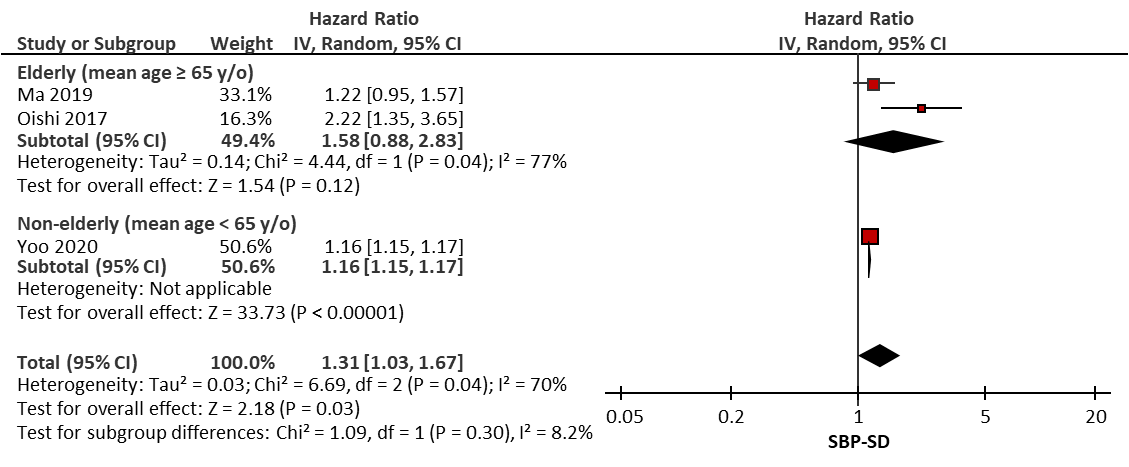
**

SBP indicates systolic blood pressure; SD, standard deviation; and IV, inverse variance. SBP-SD indicates SD of SBP. The forest plot was produced with RevMan 5.4 (Cochrane, UK) using the inverse variance method with random effects.

**Figure S15. Subgroup analysis between DBP-CV and all-cause dementia risk according to the mean age of participants in included studies**

**
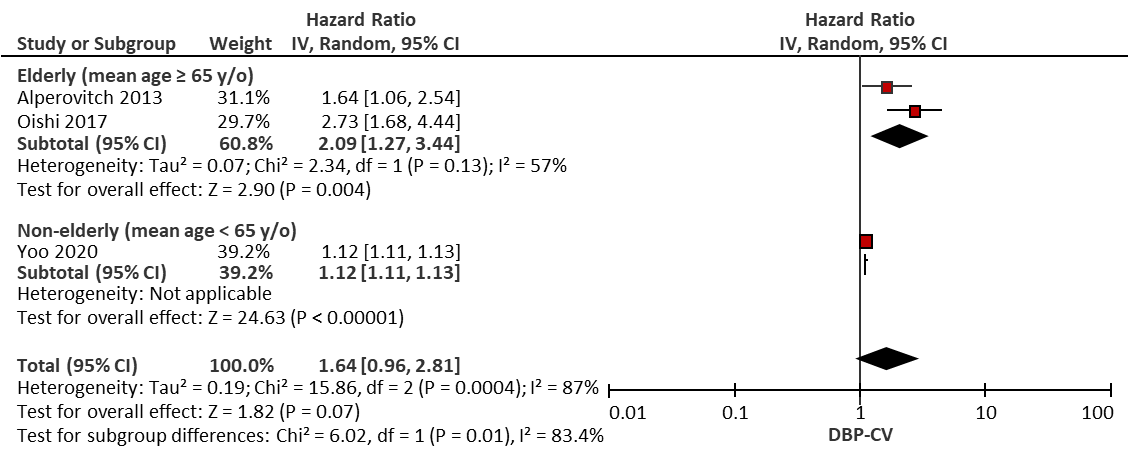
**

DBP indicates diastolic blood pressure; CV, coefficient of variation; and IV, inverse variance. DBP-CV indicates CV of DBP. The forest plot was produced with RevMan 5.4 (Cochrane, UK) using the inverse variance method with random effects.

**Figure S16. Sensitivity analysis between SBP-CV and all-cause dementia risk by leave-one-out meta-analysis**

**
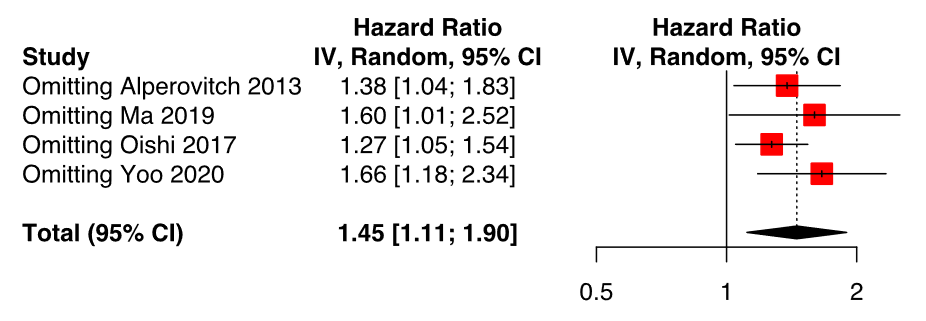
**

SBP indicates systolic blood pressure; CV, coefficient of variation; and IV, inverse variance. SBP-CV indicates CV of SBP. The forest plot was produced with the *meta* R package using the inverse variance method with random effects.

**Figure S17. Sensitivity analysis between SBP-SD and all-cause dementia risk by leave-one-out meta-analysis**

**
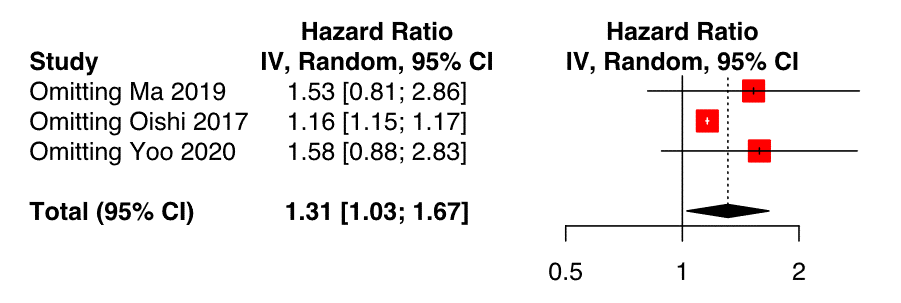
**

SBP indicates systolic blood pressure; SD, standard deviation; and IV, inverse variance. SBP-SD indicates SD of SBP. The forest plot was produced with the *meta* R package using the inverse variance method with random effects.

**Figure S18. Sensitivity analysis between DBP-CV and all-cause dementia risk by leave-one-out meta-analysis**

**
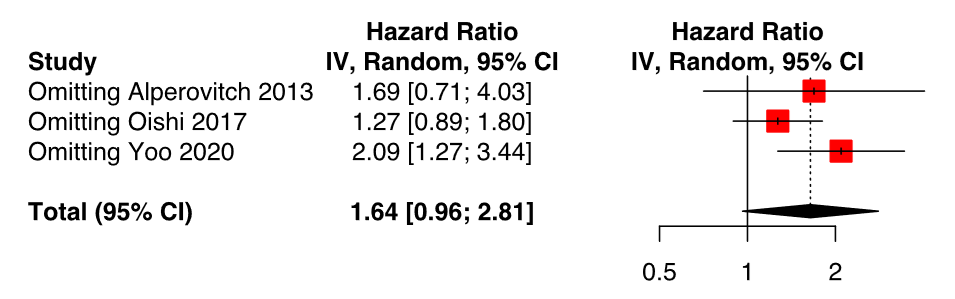
**

DBP indicates diastolic blood pressure; CV, coefficient of variation; and IV, inverse variance. DBP-CV indicates CV of DBP. The forest plot was produced with the *meta* R package using the inverse variance method with random effects.

**Figure S19. Funnel plots representing publication biases for the analysis with significant results**


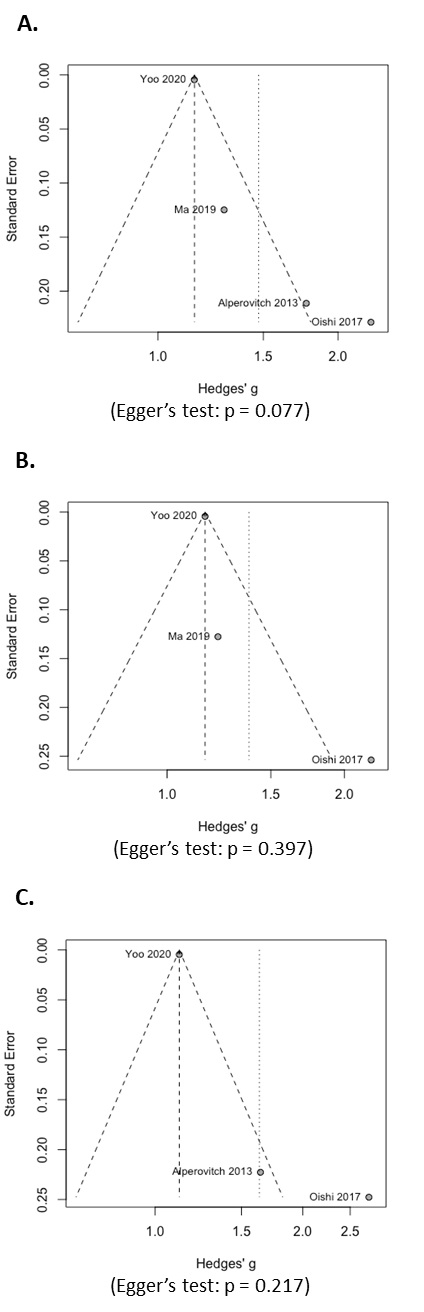


Funnel plots of hazard ratios for all-cause dementia risk attributable to SBP-CV (A), SBP-SD (B), and DBP-CV (C) were produced using the *metafor* R package, and symmetry was examined with Egger’s test. Since no significance was observed in Egger’s test, no further trim-and-fill procedure was conducted. SBP indicates systolic blood pressure; DBP, diastolic blood pressure; CV, coefficient of variation; and SD, standard deviation. SBP-CV indicates CV of SBP; similar expressions apply to SBP-SD and DBP-CV.
